# Health promotion services for lifestyle development within a UK hospital – Patients' experiences and views

**DOI:** 10.1186/1471-2458-8-284

**Published:** 2008-08-13

**Authors:** Charlotte L Haynes

**Affiliations:** 1 Clinical Effectiveness Unit, Stockport NHS Foundation Trust, Cheshire, UK

## Abstract

**Background:**

UK public health policy requires hospitals to have in place health promotion services which enable patients to improve their health through adopting healthy behaviours, i.e. health education. This study investigated hospitalised patients' experiences of health education for smoking, alcohol use, diet, physical activity, and weight, and their views concerning the appropriateness of hospitals as a setting for the delivery of health education services.

**Methods:**

Recently discharged adult hospital patients (n = 322) were sent a questionnaire asking about their smoking, alcohol use, diet, physical activity, and weight. For each of these risk factors, participants were asked whether they agreed with screening for the risk factor, whether they received health education, whether it was "helpful", and if they wanted to change their behaviour. Participants were also asked a set of general questions concerning health education within hospitals.

**Results:**

190 patients responded (59%). Over 80% agreed with screening for all risk factors. 80% of smokers, 52% consuming alcohol above recommended limits, 86% of obese, 66% consuming less than five fruit and vegetables a day, and 61% of physically inactive participants wanted to change their respective behaviour. However only a third reported receiving health education. While over 60% of patients wanted health education around discharge, the majority of those receiving health education did so at admission. The majority agreed that "hospital is a good place for patients to receive" health education (87%) and that "the hospital should provide patients with details of community organisations that provide" health education (83%). Only a minority (31%) reported a preference for health education from their GP instead of hospital.

**Conclusion:**

While the delivery of health education to patients within hospital was poor, hospitals are viewed by patients as an appropriate, and in some cases preferred setting for the screening of risk factors and delivery of health education.

## Background

There is increasing pressure on hospitals to deliver health promotion services for healthy lifestyles to patients [1-4]. The Ottawa charter for health promotion, 1986 provides a broad definition of health promotion as *"the process of enabling people to increase control over, and to improve, their health" *[5]. This can only be achieved through coordinated action from governmental and nongovernmental organisations, the health, social and economic sectors, voluntary organization, local authorities, industry, the media and communities to achieve "*healthy public policy*" [5] which creates environments supportive of health, (such as safe walking and cycling routes), supporting people in developing personal skills (by providing information on healthy lifestyles and disease management, enhancing health literacy and life skills), reorienting health services so that they focus on preventing disease/encouraging health in addition to curing existing conditions [5] and strengthening community action by involving communities in socio-political activities that impact on their own health [5,6].

The principles and practice of health promotion specifically within hospitals has been further developed in The Budapest Declaration on Health promoting Hospitals, 1991 [7] and The Vienna Recommendations on Health Promoting Hospitals, 1999 [8]. These recommendations are embodied in the guidelines developed by the World Health Organisation Health Promoting Hospitals (WHO HPH) project/network [3,4]. One of the core aims of the WHO HPH network is to facilitate change within the quality management of hospitals, with health promotion a core quality dimension [9]. To this end the WHO HPH network has recently developed a strategic and quality framework for health promoting hospitals [4]. This highlights areas which all health promoting hospitals should have as part of their quality development strategy that would benefit key stakeholders: patients, staff and communities (see table 1 for the development strategy aimed at patients). The first three strategies (PAT 1 to PAT 3) should be delivered by all health promoting hospitals as they aim to further develop the health promoting quality of the hospital services and setting, whereas strategies PAT 4 through to PAT 6 are viewed as additional health promotion strategies which hospitals can offer.

**Table 1:** Core health promotion strategies aimed at patient in Health Promoting Hospitals [4]

Health promotion by ....		Health promotion for Patients
Health promoting quality development of treatment care by..........	Empowerment of stakeholders for health promoting self-reproduction/self management	PAT 1: Health promoting living in the hospital for patients
	Empowerment of stakeholders for health promoting co-production	PAT 2: Health promoting co-production of patients in treatment
	Health promoting & empowering hospital setting for stakeholders	PAT 3: Health promoting hospital setting patients
Strategic positioning	Empowering illness management (patient education) for stakeholders	PAT 4: Health promoting illness management for patients
	Empowering lifestyle development (health education) for stakeholder	PAT 5: Health promoting lifestyle development for patients
	Participation in health promoting & empowering community development for stakeholders	PAT 6: Health promoting community setting for patients

Six strategies have also been developed for staff and communities, resulting in 18 HPH core strategies. The first three strategies are quality development strategies and the strategic positioning rows refer to additional health promotion strategies that hospitals can offer.

Key to the concept of health promotion is "empowerment" of individuals, social groups and communities: *"a process through which people gain greater control over decisions and actions affecting their health*" [10]. Health promotion interventions within healthcare can empower people through "self-reproduction": supporting patients to take responsibility for self-care physically, mentally and socially; through "co-production": collaboration of the patient in their therapy; empowering health promotion services for illness management (usually part of integrated care and beyond the hospital boundaries); and empowering health promotion services for lifestyle development: people are empowered to live healthy lives by preventing risk (e.g. not smoking or excessive alcohol intake) or enhancing lifestyles (e.g. becoming more physically active). This latter aspect of empowerment relates to the concept of "health education" which can be defined as

"*an activity that seeks to inform the individual on the nature and causes of health/illness and that individual's personal level of risk associated with their lifestyle-related behaviour.... [it] seeks to motivate the individual to accept a process of behavioural-change through directly influencing their value, belief and attitude systems, where it is deemed that the individual is particularly at risk or has already been affected by illness/disease or disability" *(p. 313 [6]).

It is the provision of health promotion services for lifestyle development (health education) to patients within hospitals which is of interest in this study (Table 1, strategy PAT 5). Specifically, the strategy for health promoting lifestyle development aims at "*improving the outcome of hospital interventions by empowering patients to build up specific health literacy (knowledge, skills and preferences) for developing and maintaining health promoting life styles*" ([4] p.30). The strategies should be viewed as a coherent programme of development as they are inextricably linked with one another. For example, for the health education strategy (PAT 5) to work optimally, patients who are empowered to self care and co-produce will be more able to take responsibility for developing and maintaining healthy lifestyles post-discharge (PAT 1 and 2). The hospital also has to be an appropriate environment for providing health education (e.g. suitable rooms; PAT 3); and empowerment to self manage diseases/impairments will often be associated with particular lifestyles (PAT 4).

In addition to the WHO HPH network strategies, public health policy in the UK now highlights the importance of providing personalised support (understanding patients' cultural and social background, etc) to patients to enable them to lead a "healthy" life (not smoking, sensible alcohol use, etc) [1]. While UK hospitals have always had elements of health promotion within routine care, the explicit demand that hospitals provide health promotion services to patients for lifestyle development, in particular focusing on smoking, alcohol misuse, obesity, diet, and exercise, is relatively new [11].

An audit of health promotion activities within 9 English hospitals revealed that while the majority of hospitalised patients were screened for smoking (94.1%) and alcohol use (83.1%), few were screened for diet (23.0%), physical activity (2.6%) or obesity (11.7%). The provision of health education was poor: an average of 22.5% of patients received health education for smoking cessation, 36.0% for alcohol misuse and very few for diet, physical activity or weight management (numbers of patients identified as requiring health education for the latter three risk factors was so low that reporting the average figures would be misleading) [12].

While the above findings present a bleak picture of health education services within hospitals, there is evidence that medical records show low levels of accuracy for the recording of such activities [13,14]. Direct observation is viewed as the "gold standard" for measuring practice; however it is not practical for exploring the provision of health education provision to hospitalised patients as these services may be delivered at any/many points during an admission. Previous research within primary care settings indicates that patients' recall of health education is more accurate than medical records [14]. It therefore appears that relying on patient recall may be the most pragmatic approach to measuring health education delivered on potentially multiple occasions and by multiple healthcare professionals.

There is evidence that approximately 50% of the English public support the NHS "taking a lead role in preventing illness and improving health" [15], and that the majority of adult hospitalised patients think hospitals should take a role in health education [16]. However, with the exception of a study by McBride, 2004, views regarding the appropriateness of hospitals as a setting for the delivery of health promotion services, including health education, have received little investigation [16].

This study aimed to provide an insight into the provision of health education for smoking, alcohol misuse, obesity, diet, and exercise within a UK hospital following UK public health policy changes highlighted above and the WHO HPH strategy for health promoting lifestyle development for patients; and to address the paucity of research on patients' views of the appropriateness/acceptability of the hospital as a setting for the delivery of health promotion services for lifestyle development.

## Methods

### Rationale

A search of the literature at the time of developing this study indicated that there was no one survey tool that adequately met the study research questions: What lifestyle development (health education) services do patients want from a hospital setting? Is the hospital a suitable setting for the delivery of health education services? And how are patients' views related to their lifestyles? A self-administered questionnaire was deemed the most appropriate tool for investigating the research questions as it could be distributed to a relatively large number of patients and allowed for anonymity, hence increasing the likelihood of honest opinions about views and experiences of health education within the hospital [17].

### Questionnaire

The following tools were adapted to assess the prevalence of smoking, alcohol misuse, obesity, physical inactivity and unhealthy diet (risk factors): Stockport Health and Lifestyle Survey [18], the "Five Shot Screening Tool": a validated test for detecting hazardous and harmful alcohol use [19], and a validated two-item dietary questionnaire [20]. As no questionnaires existed which addressed patients' attitudes to health education in hospitals a semi-structured interview was developed, based on face validity, by the researcher and steering group (patient representatives, hospital management, nursing, clinician and academic representatives). This was undertaken to the point of data saturation: 10 hospital patients differing in age, gender and ethnicity. Following content analysis of the interviews and adaptation of the aforementioned existing tools, the questionnaire was designed. The steering group and hospital patients provided comments on the coherence and legibility of the questionnaire to ensure that it adhered to the principles of good survey design [17]. The questionnaire was finalised following a pilot phase in which 19 recently discharged patients completed the questionnaire.

For the purposes of the study, the term health promotion rather than "health education" was used throughout the questionnaire as the former term was deemed more familiar to the public and there were concerns that "education" would be interpreted as reflecting a "teacher-pupil" relationship rather than a collaborative relationship where the patient plays an active role in their lifestyle development. "Health promotion" was defined as any action taken by a member of staff at the hospital to enable the patient to take control over aspects of their lifestyle that may have a negative effect on their health. Actions included verbal and written advice, medications and referral to specialists/services that aim to change unhealthy behaviours into healthy behaviours (e.g. quitting smoking). While patients were being asked their views on "health promotion", given that the definition provided more accurately reflects "health education", the latter term will be used throughout the paper.

The questionnaire followed the same format for each risk factor (smoking, alcohol, diet, exercise, and weight). Patients were asked whether they agreed that "all adult patients should be asked about their *risk factor*" (**screening agreement**). Responses were on a five point likert scale ranging from "strongly agree" to "strongly disagree". Patients were then asked whether anyone at the hospital asked them about the risk factor (**screening**). For example "During your hospital admission were you asked about the amount of alcohol you drink?" Participants could answer "yes" or "no". Measurements of height and weight were not asked about as this does not necessarily indicate that body mass index (BMI) was calculated. Information was then gathered on whether the patient has a risk factor (**risk factor prevalence**). They were then asked whether they wanted to change their "risky" behaviour (**change**). The options were "yes" or "no". Information on the type of health education delivered within the hospital was gathered. This could range from leaflets, verbal advice, referral to a specialist/service. Participants were asked to circle the correct option(s) and there was a free text option for describing any additional health education delivered. Patients were asked who delivered the health education and to rate the "**helpfulness**" of the health education on a likert scale from 1 ("not at all helpful") to 5 ("very helpful"). Patients were also asked a set of general questions relating to the appropriateness of health education within hospitals.

### Participants

Participants for this study were selected on the basis that they were from the adult (≥ 17 years old) hospitalised and day-case patient populations of one Acute Trust. A routine was established in which all patients discharged alive between January and March 2006 were identified within one week of their discharge from twelve wards representing surgery and medicine. Patients who were terminally ill were excluded from this study as it was deemed inappropriate to deliver health education to these patients.

A total of 322 people were identified and sent a questionnaire within a maximum of one month of their discharge. To optimise return-rate patients were sent a pre-letter, pre-paid return addressed envelopes along with the questionnaire, and two reminders sent 1 week and 2 weeks following the questionnaire.

### Analyses

An Excel workbook was developed for recording all data. Analyses were performed using StatsDirect Version 2.4.5 and SPSS 15.0. Patients who returned the questionnaire are described as "responders" and those who did not return the questionnaire "non-responders". Differences in age between responders and non-responders were assessed by one-way ANOVA with gender as a factor. Mann-Whitney U tests were employed to assess differences in length of stay (LoS) and Index of Multiple Deprivation (IMD) scores [21] as data were distributed non-parametrically. IMD scores provide an indicator of deprivation based on income, employment status, self-reported health and disability, educational level, housing, living environment and crime based on geographical area (the small area level). The higher the score, the greater the level of deprivation. Proportion differences for screening of smoking and alcohol use, smoking behaviour and alcohol misuse were calculated for responders versus non-responders based on findings in patients' case notes. All reported significance values are two-tailed. In order to describe the responses to all questions, these were subject to descriptive statistics: proportions, proportions ± 95% confidence interval (CI) and tests for proportion differences where appropriate. SCREENING AGREEMENT questions were also subject to the Friedman test to examine whether responses were similar across the four risk factors (smoking, diet, alcohol and exercise).

This study received Local Research Ethics approval.

## Results

### Demographics

One hundred and three females and eighty-seven males returned the questionnaire (59% response rate). The mean age and median length of stay for responders and non-responders are reported in table 2. There was a trend towards male non-responders being younger than female non-responders and all responders. There were no significant differences in the median length of stay or socio-economic status (IMD score) between the groups, nor in the proportion of responders and non-responders who were (according to their medical case notes) screened for smoking and alcohol, or identified as misusing alcohol (see table 3). There were however significantly more smokers (according to case note information) amongst non-responders compared to responders.

**Table 2:** Demographics

Age*	**Range (years)**	**Total (mean ± SE)**	**Females (mean ± SE)**	**Males (mean ± SE)**
Responders	17 – 96	57.0 ± 1.3	56.3 ± 2.0	57.9 ± 1.9
Non-responders	17 – 98	52.9 ± 2.0	56.4 ± 3.0	49.4 ± 2.6

**Length of stay (LoS)**	**Range (days)**	**Total (median)**	**Females (median)**	**Males (median)**

Responders	1 – 50	4	4	5
Non-responders	1 – 197	4	4	4

**IMD Score**	**Range**	**Total (median)**	**Females (median)**	**Males (median)**

Responders	2.36 – 66.35	14.77	15.09	14.71
Non-responders	2.64 – 76.39	17.98	17.29	18.31

*Main effect of responder F_(1) _= 3.185, P = 0.08; main effect of gender F_(1) _= 1.279, P = 0.26; responder × gender interaction F_(1) _= 3.328, P = 0.07. IMD = Index of multiple deprivation

**Table 3:** Differences in the prevalence of smoking and alcohol misuse between responders and non-responders

**Audit findings**	**Responder**	**Non-responder**
Screened for smoking	168/190 (0.88; CI = 0.83 to 0.93)	120/133 (0.90; CI = 0.84 to 0.95)
Screened for alcohol use	145/190 (0.76; CI = 0.70 to 0.82)	101/133 (0.76 CI = 0.68 to 0.83)
Identified as a smoker*	37/167 (0.22; CI = 0.16 to 0.30)	50/119 (0.42; CI = 0.33 to 0.51)
Alcohol consumption above recommendations	26/138 (0.19; CI = 0.13 to 0.26)	25/85 (0.29; CI = 0.20 to 0.40)

*Proportion difference = 0.199, 95% CI = 0.090 to 0.306, P < 0.0005

### Questionnaire findings

#### Screening

The majority of responders reported that they were asked by a member of the hospital staff whether they smoked (156/183; 95% CI = 0.79 to 0.90) and they were asked about their alcohol use (115/188; 95% CI = 0.54 to 0.68). However only about a quarter were asked about their usual diet (46/184; 95% CI = 0.19 to 0.32) and physical activity (42/175; 95% CI = 0.18 to 0.31).

#### Risk factor prevalence and health education delivered

Additional file 1 reports the proportion of responders identified by the questionnaire as having a risk factor, and of those with a risk factor, the proportion receiving any type of health education, plus details of the form health education took for each risk factor. Of the 20.5% respondents identified as smokers, 44.4% reported receiving some type of health education. The proportion of patients identified as misusing alcohol according to self-reported weekly consumption compared to the Five shot tool is not significant (proportion difference = -0.019, 95% CI = -0.11 to 0.08 P = 0.70). Between 21% and 29% of these patients received health education for alcohol misuse. While the majority of respondents consumed less than five portions of fruit and vegetables a day and were physical inactive, only 11.9% and 15.4% received health education for diet and physical activity respectively. Nearly half of respondents were either obese or overweight, but very few received any form of health education concerning weight loss. In total sixty-three patients reported receiving health education for at least one risk factor during their hospital episode (i.e. 33.2% of respondents). Forty-four received health education for one risk factor only, eleven for two risk factors, six for three risk factors and two received health education for four risk factors.

**Figure 1:**
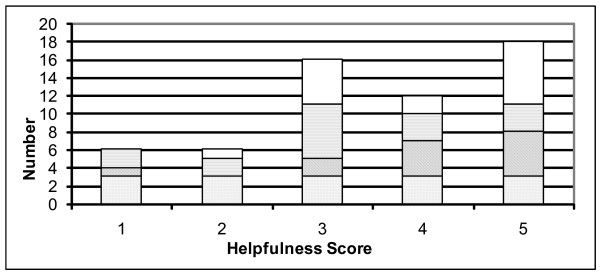
**Ratings of "helpfulness" for health promotion delivered**. A score of 1 indicated that the health promotion was "not at all helpful" and a score of 5: "very helpful". White fill: Exercise, Horizontal lines fill: Diet, Diagonal lines fill: Alcohol, Grey dots fill: Smoking.

Figure 1 reports the "helpfulness" ratings for the health education delivered. 51.7% of patients reported that the health education they received was helpful (score of 4 or 5), 27.6% appear undecided (score of 3) and 20.7% viewed the health education services as unhelpful (score of 1 or 2).

#### Change

**Figure 2:**
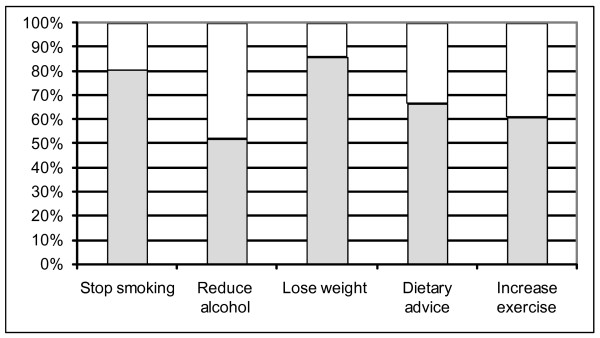
**Percentage of responders who wanted to change a risk factor**. Figures are based on identified need. Grey fill: Yes, White fill: No.

Figure 2 shows the percentage of participants who reported that they would like advice concerning how to change a risk factor (only for those with evidence of the risk factor). The majority of smokers wanted to quit smoking. Based on self-reported alcohol consumed in the past week, 16/31 identified as harmful/hazardous drinkers reported that they wanted to reduce their alcohol intake. On the basis of the Five shot tool 25/48 participants categorised as potentially misusing alcohol said they wanted to reduce their alcohol intake. Of those who consumed alcohol within recommended weekly limits (100) only 9 wanted to reduce their alcohol intake; and only 2/140 participants categorised as having no alcohol problem according to the Five shot tool wanted to reduce their alcohol intake.

While approximately half of all respondents wanted to reduce their weight, when desire to lose weight was related to BMI, 86% of obese individuals (BMI >30 Kg/m^2^; see figure 2), 78% of overweight individuals (BMI 26–30 Kg/m^2^), 28% of those with a normal BMI (18.5–25 Kg/m^2^) and none of the underweight participants (BMI <18.5 Kg/m^2^) wanted to lose weight.

Significantly more people who consumed less than five portions of fruit and vegetables a day wanted dietary advice during their hospital admission compared to those who consumed five portions a day (67% and 36% respectively, chi-square test using continuity correction, P = 0.0012).

There was no difference in the proportion of patients who wanted to increase exercise when assessed according to whether patients engaged in the recommended 30 minutes of moderate physical activity five times a week or not (55.1% and 61.3% respectively, chi-square test using continuity correction, P = 0.397).

### Appropriateness of health education within hospitals

#### Screening agreement

**Figure 3:**
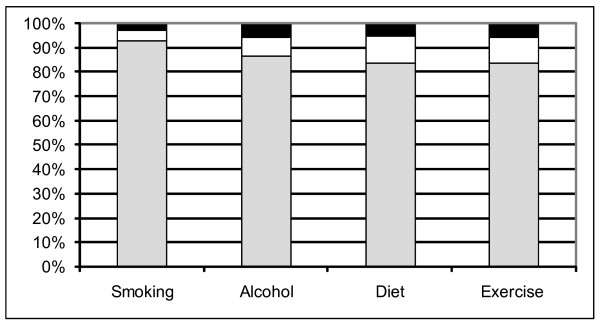
**Percentage of responders agreeing with the statement that all patients should be asked about risk factors**. Grey fill: Agree, White fill: Undecided, Black fill: Disagree.

Inspection of figure 3 reveals that the majority of participants "strongly agreed"/"agreed" with all adult hospital patients being asked about their smoking, alcohol use, diet and exercise. The overall Friedman test statistic was significant (F = 31.94, P < 0.0001). Patients agreed significantly more with screening for smoking compared to screening for all other risk factors (P < 0.0001 for smoking compared to each remaining risk factor) and with screening for alcohol compared to exercise (P < 0.02).

#### Timing of health education

**Figure 4:**
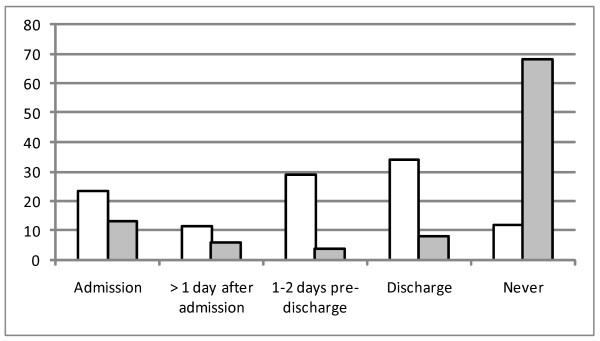
**When patients want health promotion and when they were delivered health promotion within the hospital**. White fill: When patients wanted to receive health education, Grey fill: When patients received health education.

Of those participants who received health education, 52/63 reported when it was delivered. The majority reported that health education was delivered at only one time-point, with only three patients reporting health education at two time points. While the majority of patients who received health education within the hospital reported that it was delivered on admission (22/52), most reported that they would like health education delivered just prior to discharge or at discharge (see figure 4). Admission was the third most popular time for receiving health education, and just after admission the least popular. A number of patients (10.9%) also reported that they did not want any form of health education during their hospital admission.

#### Hospital setting

The majority of participants agreed that hospital was a "good place" to receive health education and that "the hospital should provide patients with details of community organisations that provide" health education (see Table 4). The largest proportion of respondents were undecided about whether they would prefer to get health education from their GP rather than hospital staff.

**Table 4:** Responses to general statements

	**Response**					
**Statement**	**Strongly agree**	**Agree**	**Undecided**	**Disagree**	**Strongly disagree**	**Total (n)**

Hospital is a good place for patients to receive HE	22.9%	63.7%	10.1%	2.8%	0.6%	179
The hospital should provide patients with details of community organisations that provide HE	21.0%	61.9%	13.6%	3.4%	0.0%	176
I would prefer HE from GP rather than hospital staff	4.5%	27.3%	44.3%	19.9%	4.0%	176
I would like the hospital to let my GP know about my smoking, diet, exercise, and alcohol intake	14.0%	40.7%	21.5%	18.0%	5.8%	172
I would like the hospital to let my GP know about the HE I received from hospital staff	10.8%	50.0%	23.5%	12.0%	3.6%	166

HE: health education (written as "health promotion" in full in the questionnaire)

Just over half of the responders would like the hospital to let their GP know about their risk factors and the health education they received while in hospital. While 23.8% did not want their GP to know about their risk factors, only 15.7% did not want the hospital to inform their GP of the health education they had received. Out of the 31 disagreeing with letting their GP know about their risk factors, 2 were smokers, 2 reported consuming alcohol above recommended limits (and 7 had positive five shot scores). Of the 10 strongly disagreeing, 3 were smokers and 2 misused alcohol (self-reported units and five shot tool). Exactly the same people who misused alcohol "disagreed"/"strongly disagreed" with their GP being told about the health education they received, but only 1 smoker (strongly) disagreed with their GP being told about the health education received.

## Discussion

The results of this survey indicate that hospital patients view hospitals as an appropriate place for the delivery of health education for all risk factors. While there was clear support for screening all adult hospital patients for risk factors, reported screening was not ideal for any of the risk factors, in particular for diet and physical activity. There was also considerable demand for health education, but little provision.

While it appears that the hospital may not be meeting UK public health requirements or international standards for health education within hospitals [1-4], some may question whether patients' recall was accurate. Patients' memory may be affected by the emotional state they were in when information was imparted, potentially resulting in attentional narrowing or state-dependent learning [22,23]. Memory may also be affected by the perceived importance of information (diagnosis is viewed as very important and treatment less so), and age-related cognitive impairments [23]. The communication style of healthcare professionals delivering health promotion services also affects recall, with patients more likely to remember medical information if healthcare professionals provide simple to follow, specific written instructions (rather than general/verbal instructions) [23]. Medical information is least likely to be recalled, and therefore acted upon, if it is delivered verbally compared to written/pictorial presentation or a combination of written and verbal presentation, yet the most frequent form health education took was verbal advice.

The questionnaire showed good criterion validity as risk factor prevalence in this sample was similar to national and local profiles for the age group: 22% of the local population are smokers and 22% are estimated to be obese [24]. In England, 87% of the population does not consume five portions of fruit and vegetables a day, 63% of men and 76% of women are physically inactive [25]; and approximately 20% of adult in-patients are expected to be hazardous or harmful drinkers [26]. The percentage of smokers wanting to quit is also similar to previous reports that more than 70% of smokers wish to quit [27]. There is however little data on the proportion of people with other risk factors who want to change their behaviour. The information on the percentage of patients wishing to reduce the amount of alcohol they drink, reduce their weight, and improve their diet and level of physical activity can be used by hospitals as the basis for developing realistic standards for the number of patients delivered health education once a risk factor has been identified. The findings indicate that hospitals should initially aim to deliver health education to a minimum of 70% of smokers and obese individuals and 50% of people misusing alcohol, consuming an unhealthy diet and physically inactive.

The timing of health education is also important to patients. While the majority felt that the time around discharge was the most appropriate period for health education, those receiving health education reported that it was usually delivered on admission. This may be because admission is the usual time for screening of risk factors. However, it is likely that this is not necessarily the optimum time for health education as patients may be in an unreceptive state due to their condition and their primary concern may be the immediate improvement in health (Haynes, 2004 unpublished).

As the purpose of health promotion is to enable people to change a behaviour, it is important to assess whether the different potential forms of health education have achieved this. This was evaluated by asking patients how "helpful" the health education services they received were. While more patients viewed the services as "helpful" than "unhelpful", given that so few patients received any form of health education, it is not possible to draw definitive conclusions concerning the "helpfulness" of the different services. It is recommended that this question remains as it could provide valuable information about health education services when they are delivered to a larger number of patients.

Given that primary care is the traditional setting for the delivery of health education it was surprising that approximately a quarter of respondents expressed disagreement with the statement *"*I would prefer health promotion [education] from my GP rather than hospital staff". While this infers that they would prefer health education from a hospital, further research is required to verify this finding (e.g. changing the statement to "I would prefer to get health education from hospital staff instead of my GP"); and to explore the reasons for this preference. For example, are hospital staff viewed as more approachable than GPs? Is there a perception of greater anonymity within a hospital, circumventing embarrassment? Although 1 in 10 respondents expressed that they never wanted health education while hospitalised, it is encouraging that only 3% disagreed with the statement that "hospital is a good place for patients to receive health promotion [education]".

Disagreement with the statements "I would like the hospital to let my GP know about my smoking, diet, exercise, and alcohol intake/the health promotion [education] I received from hospital staff" indicates that there is an objection to the sharing of information between hospitals and GPs. However, the use of the word "like" may be the reason, and further research is required to clarify whether there is an actual objection. If there is a true objection to communication between hospitals and GPs, it may be difficult to justify automatically informing a patient's GP of their patient's risk factors and health education delivered, and hospitals may need to ensure that they have patient consent for sharing this type of information. However an argument can be made for the transfer of information as being in the public interest and part of continuing care, and therefore not subject to the need for informed patient consent [28]. With the drive towards greater collaboration/communication between services, and the creation of electronic patient records available to all health care providers, these findings are of concern. Reasons for such an objection also require clarification. There was no evidence that disagreement with the sharing of information was related to the presence of risk factors.

### Study Limitations

There were some limitations to this study. The sample included patients from only one hospital and results may therefore not be generalisable to other hospital populations. The trend towards fewer young men responding to the questionnaire indicates that the views of this group were not expressed. Young men are less likely than other populations to respond to questionnaires [29]; and it may be that other methods such as focus groups are more appropriate for investigating this group's preferences for health promotion services for lifestyle development. The views of this population need to be explored as young men consume more alcohol and less fruit and vegetables than older adults, and are more likely to be smokers [25]; hence they are at risk of developing chronic diseases. The finding that smokers were less likely to respond to the questionnaire may have repercussions on the findings concerning the receptivity of patients to health education. Previous research has indicated that smokers are less likely to believe that "the way people live affects their health" [16], suggesting they may be less responsive to health education, and that hospitals may face additional challenges in successfully delivering health education to smokers.

While there was a reasonable response rate to the questionnaire, the poor provision of health education meant that statistical analysis of some questionnaire items was limited by small sample sizes. Improvements in the provision of health education within hospitals and/or larger sample sizes would improve the situation. Some questionnaire items require minor changes in wording to allow for definitive conclusions concerning patients' views on sharing information between healthcare sectors and preferences for hospitals over GPs for health education delivery.

## Conclusion

The findings of this study should encourage hospitals to improve their health promotion services. Hospital healthcare professionals should be made aware that approximately a quarter of patients may in fact prefer health education from a hospital setting rather than their GP, that there is a demand for health education, and agreement with screening of all risk factors. This information may allay concerns that some risk factors are too sensitive to address, and challenge the view that lack of receptivity from patients is an obstacle to delivering health education [30,31]. Health education is however not for everyone, with approximately 1 in 10 patients choosing not to receive health education for risk factors while hospitalised, and staff need to ensure that they respect this desire. The (apparently substantial) challenge now facing UK hospitals, is how will they meet their patients' demand for health education? Guidance on how to embed health promotion services within hospitals has been provided by the WHO HPH [3,4]. While there is some controversy concerning the "success" of this project/network [32-35], there are examples of successful health promotion projects within hospitals and some reports of hospitals becoming health promotion settings [36-38], lessons from which, UK hospitals would be wise to learn from.

## Competing interests

The author declares that they have no competing interests.

## Authors' contributions

CH was involved in the conception and design of the project. CH collected, analysed and interpreted the data. CH wrote the entire article.

## Pre-publication history

The pre-publication history for this paper can be accessed here:



## Supplementary Material

Additional file 1 **Table – Proportion of patients with a risk factor and proportion delivered health promotion**. data for Table. Click here for file
