# Supplementary material for: Health promotion services for lifestyle development within a UK hospital – Patients' experiences and views
Source: BMC Public Health. 2008 Aug 13;8:284. doi: 10.1186/1471-2458-8-284 (PMC2527563; doi:10.1186/1471-2458-8-284)
Supplement: Additional file 1 — Table – Proportion of patients with a risk factor and proportion delivered health promotion. data for Table. [file 1471-2458-8-284-S1.doc]

Table - Proportion of patients with a risk factor and proportion delivered health promotion

|  | Risk factor identified | Health promotion delivered | **Type of health promotion delivered** | | | | |
| --- | --- | --- | --- | --- | --- | --- | --- |
|  | Proportion (95% CI) | Proportion (95% CI) | Leaflet | Verbal advice | Contact information given (GP or nurse) | Drug treatment | Referral to a specialist |
| Smoker | 36(29)*/176 (0.15 to 0.27) | 16/36 (0.28 to 0.62) | 4 | 11 | 1 | 7 | 0 |
| Misusing alcohol – self report units | 31/131 (0.17 to 0.32) | 9/31 (0.14 to 0.48) | 4 | 3 | 0 | 0 | 5 |
| Misusing alcohol – Five shot tool | 48/188 (0.19 to 0.32) | 10/48 (010. to 0.35) |
| Consuming less than 5 portions of fruit/vegetable a day | 151/183 (0.76 to 0.88) | 18/151 (0.07 to 0.18) | 6 | 11 |  |  |  |
| Participating in less than 30 minutes of moderate intensity physical activity 5 times a week. | 123/175 (0.63 to 0.77) | 19/123 (0.10 to 0.23) | 6 | 19 |  |  | 2 |
| Obese (BMI ≥30) | 44/183 (0.18 to 0.31) | 5/44 (0.04to 0.25) | 1 | 3 | 1 |  |  |
| Overweight (BMI >25<30) | 41/183 (0.17 to 0.29) | 2/41 (0.01 to 0.17) |  |  | 1 |  | 1 |

Legend: * Numbers shown are those who have been designated as smokers by the researcher based on the finding that in the questionnaire they reported quitting smoking <28 days ago. Numbers in brackets reflect those categorised according to self-reported “current smoker” at the time of completing the questionnaire.
